# Vascular Endothelial Growth Factor Receptor 2: Molecular Mechanism and Therapeutic Potential in Preeclampsia Comorbidity with Human Immunodeficiency Virus and Severe Acute Respiratory Syndrome Coronavirus 2 Infections

**DOI:** 10.3390/ijms232213752

**Published:** 2022-11-09

**Authors:** Tashlen Abel, Jagidesa Moodley, Olive P. Khaliq, Thajasvarie Naicker

**Affiliations:** 1 Women’s Health and HIV Research Group, Department of Obstetrics & Gynaecology, School of Clinical Medicine, College of Health Sciences, University of KwaZulu-Natal, Durban 4000, South Africa; 2 Department of Paediatrics and Child Health, Faculty of Health Sciences, The University of the Free State, Bloemfontein 9300, South Africa; 3 Optics and Imaging Centre, Doris Duke Medical Research Institution, College of Health Sciences, University of KwaZulu-Natal, Durban 4000, South Africa

**Keywords:** preeclampsia, pregnancy, human immunodeficiency virus, severe acute respiratory syndrome coronavirus 2, microrna, vascular endothelial growth factors

## Abstract

This review explored the role of vascular endothelial growth factor receptor-2 (VEGFR-2) in the synergy of preeclampsia (PE), human immunodeficiency virus (HIV), and severe acute respiratory syndrome coronavirus 2 (SARS-CoV-2) infections. Downregulation of VEGFR-2 in PE promotes endothelial dysfunction and prevents endothelial cell (EC) migration, proliferation, and differentiation. The HIV-1 accessory protein, tat (trans-activator of transcription), prevents VEGFR-2 signaling via the vascular endothelial growth factor A (VEGF-A) ligand. Combined antiretroviral therapy (cART) may cause immune reconstitution, impaired decidualization, and endothelial injury, thus may be a risk factor for PE development. The VEGF/VEGFR-2 interaction may be associated with SARS-CoV-2-related pulmonary oedema. Endothelial dysfunction and heightened inflammation are both associated with PE, HIV, and SARS-CoV-2 infection; therefore, it is plausible that both characteristics may be exacerbated in the synergy of these events. In addition, this review explored microRNAs (miR) regulating VEGFR-2. An overexpression of miR-126 is evident in PE, HIV, and SARS-CoV-2 infection; thus, modulating the expression of miR-126 may be a therapeutic strategy. However, the involvement of microRNAs in PE, HIV, and SARS-CoV-2 infection needs further investigating. Since these conditions have been evaluated independently, this review attempts to predict their clinical manifestations in their synergy, as well as independently; thereby providing a platform for early diagnosis and therapeutic potential in PE, HIV, and SARS-CoV-2 infection.

## 1. Introduction

Worldwide, HIV-1 infection and hypertensive disorders of pregnancy (HDP) remain the major indirect and direct causes of maternal deaths, respectively. Low- and middle-income countries (LMIC), such as South Africa (SA), experience higher maternal mortality rates than high-income countries [1]. Preeclampsia (PE), is an HDP that is one of the leading direct causes of maternal morbidity and mortality, complicating 5–7% of pregnancies globally with a prevalence of 12% in the South African province of KwaZulu-Natal (KZN) [2,3,4]. In Eastern and Southern Africa, the female population accounted for 63% of all new HIV infections in 2021 [5]. Despite combined antiretroviral therapy (cART) improving life expectancy and reducing vertical transmission, its use is associated with increased PE development, disproportionately higher female mortality rates, low birth weight, and preterm births [6,7]. Furthermore, the SA healthcare system has been under enormous strain due to the emergence of the severe acute respiratory syndrome coronavirus 2 (SARS-CoV-2) [8]. This virus affected the globe and was declared a pandemic in March 2020 by WHO [9]. The rapid spread of the virus led to strict rules and regulations that resulted in a complete lockdown. During the COVID-19 (coronavirus disease 2019) lockdown, maternal deaths increased by 30% in SA [8]. Reportedly, the duality of PE and HIV infection increases susceptibility to SARS-CoV-2 infection and the triad of diseases impacts inflammatory responses and endothelial function [10,11]. It is vital to ascertain the conceptual framework underlying the interaction of PE, HIV infection, cART usages, and SARS-CoV-2 infection in order to prevent maternal deaths.

The vascular endothelial growth factor receptor 2 (VEGFR-2) is found on vascular endothelial cells (EC) and their embryonic precursors including placental trophoblast cells and hence within placental villi [12,13]. It has the ability to bind all VEGFs except VEGF-B and the placental growth factor (PlGF), when its co-receptor, neuropillin-1 (NRP-1), is bound [14]. Vascular endothelial growth factor receptor 2 is involved in various angiogenic indicators by influencing mitogenic cell signaling and the migratory activity of EC [15]. Moreover, VEGFR-2 is reportedly decreased in PE compared to normotensive pregnancies and dysregulated in HIV and SARS-CoV-2 infection [16,17]. However, the involvement of VEGFR-2 in the triad of PE, HIV infection with cART usage, and SARS-CoV-2 infection remains to be elucidated. Additionally, microRNAs (miRNA) that regulate VEGFR-2 are reportedly dysregulated in PE, HIV infection, and SARS-CoV-2 infection which is indicative of its potential involvement in the pathogenesis of these complications [18,19]. This review examines the role and regulation of VEGFR-2 in PE, HIV infection, and SARS-CoV-2 infection, as well as potential therapeutic strategies.

## 2. Vascular Endothelial Growth Factors and Receptors in Placental Angiogenesis

**Figure 1:**
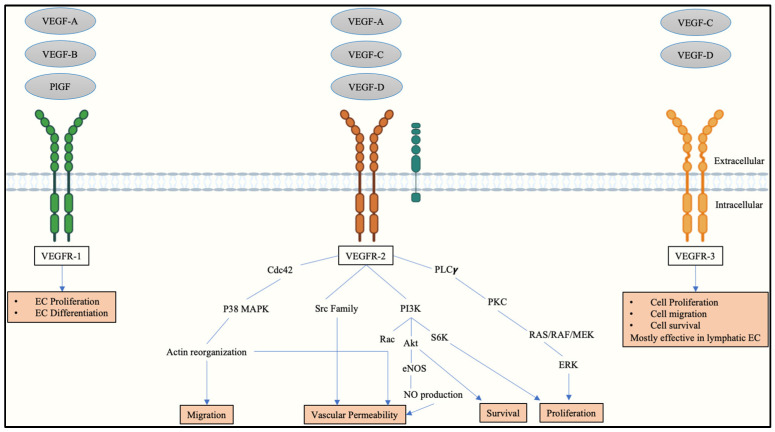
Schematic representation of the VEGF family and the signaling pathway of VEGFR-2. Abbreviations: vascular endothelial growth factor receptor-1 (VEGFR-1), vascular endothelial growth factor receptor-2 (VEGFR-2), vascular endothelial growth factor receptor-3 (VEGFR-3), vascular endothelial growth factor-A (VEGF-A), vascular endothelial growth factor-B (VEGF-B), vascular endothelial growth factor-C (VEGF-C), vascular endothelial growth factor-D (VEGF-D), placental growth factor (PlGF), neuropilin-1 (NRP-1), endothelial cell (EC), Mitogen-activated protein kinase (MAPK), phospholipase-Cγ (PLCγ), Src (proto-oncogene tyrosine-protein kinase sarcoma), protein kinase C (PKC), Rat sarcoma virus (RAS), Rapidly Accelerated Fibrosarcoma (RAF), Extracellular-signal-regulated kinase (ERK), Phosphoinositide-3 kinase (PI3K), Ribosomal protein S6 kinase (S6K), protein kinase B (Akt), endothelial nitric oxide synthase (eNOS), nitric oxide (NO).

The homeostatic balance between local and systemic pro- and anti-angiogenic factors is essential for a healthy and successful pregnancy [20]. Angiogenesis is mainly regulated by VEGF (vascular endothelial growth factor) which consist of six isoforms, namely VEGF-A, VEGF-B, VEGF-C, VEGF-D, and PlGF (Figure 1) [21]. These mediators bind to and activate VEGFR-1, VEGFR-2, and VEGFR-3 [22]. Specifically, VEGF-A binds to VEGFR-1 and VEGFR-2, VEGF-B, and PlGF bind to VEGFR-1 whilst VEGF-C and VEGF-D bind to VEGFR-3 and influence the lymphangiogenic pathway [22]. In addition, VEGF-D is also able to bind to VEGFR-2 [22]. Although VEGFR-1 possesses a greater affinity for VEGFs, VEGFR-2 has a greater tyrosine kinase activity and has the potential to bind all VEGF proteins when its co-receptor, NRP-1, is bound, except VEGF-B and PlGF, [14]. Abnormal placentation is one of the most common placental pathologies identified in pregnancy complications in animals and humans, and is associated with adverse maternal and perinatal outcome [23,24]. In humans, abnormal placentation is characteristic of PE, the most commonly diagnosed HDP (Figure 1) [25].

## 3. Vascular Endothelial Growth Factor Receptor-2 in a Physiologically Stable Pregnancy

Vascular endothelial growth factor is a potent angiogenic factor that plays a pivotal role in various physiological and pathological environments [26]. It is involved in embryo implantation through enhancement of embryo development, improving endometrial receptivity, and facilitating the interactions between the developing embryo and the endometrium [26]. Dysregulated expression of VEGF is correlated with reproductive failure which includes recurrent implantation failure and recurrent miscarriage [26].

Vascular endothelial growth factor receptor-2 is expressed on hemangioblasts with mesodermic origin at day 7.5 of gestation and is involved in the initiation of vasculogenesis via its effect on their migration, differentiation into EC, and the formation of vascular islands in the yolk sac [27]. Neufeld et al. reported that the inactivation of the murine gene that encodes for VEGFR-2 in homozygotic animals led to the death of embryos on days 8 to 9 [28]. Embryonic death occurred due to the prevention of EC differentiation thereby blocking the development of the vascular system [28]. Hence, VEGFR-2 plays a pivotal role in vasculogenesis during embryonic development.

Investigations utilizing animal models revealed that uterine decidual angiogenesis is stimulated by VEGFR-2 during early pregnancy [29]. VEGF-dependent pathways are evident in the ovary, uterus, and embryo; impairment to the physiological functioning of VEGF in any of these areas might prevent physiologically normal pregnancy development [29]. Both VEGF and VEGFR-2 are expressed during the formation of the corpus luteum (CL) in rodents, nonhuman primates, as well as humans [30,31]. Using angiogenic inhibitors, such as anti-VEGF antibodies and VEGFR-2 blocking antibodies, it was reported that the VEGF/VEGFR-2 signaling pathway is involved in angiogenic regulation in corpora lutea [30,32,33,34]. Embryonic development is enhanced by VEGF through improvement in endometrial receptivity to the implanted embryo [26]. Disruption to the VEGF/VEGFR-2 signaling pathway that leads to abnormal peri-implantation angiogenesis may cause early miscarriage [35,36].

A study conducted in rats concluded that in the early and middle stages of pregnancy, VEGF expressed in the luteal cells contribute to luteal angiogenesis, CL formation, and progesterone production [33,37]. Since the expression of VEGF is heightened in the mature CL during late stages of pregnancy followed by a sudden decline near the end of pregnancy, VEGF may highly influence the regulation of luteal function during the early and later stages of pregnancy [38]. VEGF appears to be essential for all stages of pregnancy because it ensures the supply of sufficient blood flow to luteal cells through luteal angiogenesis, maintenance of the luteal vascular function, or both [39].

The autophosphorylation site Y1175 of VEGFR-2 is essential for VEGF-A-induced proliferation in EC [40]. Stimulation of VEGFR-2 by VEGF-A activates signaling pathways such as phospholipase-Cγ (PLCγ)/protein kinase C (PKC) and RAS (Rat sarcoma virus)/RAF (Rapidly Accelerated Fibrosarcoma)/ERK (Extracellular-signal-regulated kinase)/MAPK (Mitogen-activated protein kinase) pathways, all of which impacts EC proliferation [13,41]. The activation of the PI3K (Phosphoinositide-3 kinase)/Akt (protein kinase B) pathway, facilitates an anti-apoptotic role in EC survival, as well as cellular migration via the activation of integrins which disrupt cell-to-cell adhesion [41,42]. Moreover, the activation of the PI3K and p38 MAPK pathways led to the formation of a complex between VEGFR-2 and adhesion molecules such as cadherins (vascular endothelial (VE)-cadherin), β-catenin, occludins, and connexin-43 [43]. This complex weakens the intercellular junctions, destabilizes the cytoskeleton of EC, and induces the formation of endothelial fenestrae [43]. Ultimately, this increases vascular permeability favoring EC migration [43,44]. Vascular permeability is further exacerbated by the activation of Akt protein kinase which stimulates the formation of endothelial nitric oxide synthase (eNOS) and nitric oxide (NO) [43].

## 4. Vascular Endothelial Growth Factor Receptor 2 in Preeclampsia

Preeclampsia is a pregnancy-related disorder characterized by new-onset hypertension (systolic blood pressure >140/diastolic blood pressure >90 mmHg) with/without proteinuria, presenting at or after 20 weeks of gestation [45,46]. In the event of new-onset hypertension in pregnant women without proteinuria, PE is characterized by haemolysis, elevated liver enzymes, and low platelet count, otherwise referred to as the HELLP syndrome [47]. Additionally, PE is divided into early-onset PE (EOPE) and late-onset PE (LOPE), diagnosed ≤33 weeks but >20 weeks and ≥34 weeks of gestation, respectively [48]. Furthermore, EOPE is primarily associated with abnormal placentation and referred to as “placental PE”, while LOPE is referred to as “maternal PE” since it may be associated with widespread maternal endothelial dysfunction and an imbalance between normal maternal perfusion and the metabolic demands of the placenta and fetus [10,48]. Although the pathogenesis of PE is yet to be fully elucidated, it is accepted that it occurs in two stages [49]. The first stage (pre-clinical stage) occurs in the first and second trimesters and involves deficient extravillous trophoblast invasion together with an absence of a physiological transformation of myometrial spiral arteries [50]. The first stage leads to maternal EC injury that precedes the clinical characteristics of PE. The clinical features of PE present in the second stage of PE development which occurs during the second and third trimesters [51]. Endothelial cell damage leads to the release of placental and maternal factors into the maternal circulation that disrupt the balance of angiogenic mediators [51]. An angiogenic imbalance that favors anti-angiogenic mediators is characteristic of PE development.

Increased circulating anti-angiogenic factors such as soluble VEGFR-1 (sVEGFR-1) and soluble endoglin (sEng) coupled with a subsequent decline in VEGFR-1 and PlGF impairs vasculogenesis and angiogenesis potentially predisposing PE development [52]. Circulating sVEGFR-1 binds and sequestrates both VEGF-A and PlGF, preventing the binding of both factors to their native receptor, VEGFR-2, which impedes EC activity and vascular development [53]. Additionally, sEng promotes apoptosis and prevents the synthesis of eNOS, thereby impairing EC migration and proliferation [54]. Improper vascular remodeling of the spiral artery in PE causes uteroplacental hypoperfusion leading to placental hypoxia which promotes an intense activation of an inflammatory response [51]. The resultant effect is an increase in the release of pro-inflammatory cytokines including interleukins (IL-1β, IL-6, IL-2, IL-8), interferon-gamma (INF-γ), tumour necrosis factor-alpha (TNF-α), and syncytiotrophoblast microparticles (STBM) which further prevents the synthesis of eNOS and therefore reduced vasodilation which is maintained by placental oxidative stress [55,56]. Ultimately, the anti-angiogenic and pro-inflammatory state of PE favors vasoconstriction primarily due to heightened systemic expression of the powerful vasoconstrictor, endothelin-1 (ET-1) which is maintained by calcium influx and vascular resistance during endothelial damage in PE [16,57]. Moreover, Lely et al. characterized PE as expressing decreased VEGFR-2 and sVEGFR-2 in maternal circulation [58].

Vascular endothelial growth factor receptor-2 is reportedly downregulated in preeclamptic placentae compared to normotensive patients [59], albeit reports are conflicting [60]. Similar to the placentae of preeclamptic patients, the mRNA expression of VEGFR-2 is downregulated in maternal serum [61] and peripheral blood mononuclear cells (PBMCs) of preeclamptic compared to normotensive patients [62]. Furthermore, VEGFR-2 is significantly downregulated in LOPE compared to EOPE [62]. This may be due to widespread maternal vascular endothelial damage evident in LOPE [10,57]. Importantly, these investigations did not report whether the placental samples were obtained before labour since labour is associated with an increased release of placental proteins, including VEGF [63]. An inversely proportional relationship exists between sVEGFR-1 and VEGFR-2 with an increase in sVEGFR-1 and a decrease in VEGFR-2 in PE [64]. The binding and sequestering of VEGF by sVEGFR-1 leads to decreased bioavailability of VEGF [42,62]. It is plausible that reduced bioavailability of VEGF leads to a decrease in VEGFR-2 as free VEGF stimulates VEGFR-2 synthesis along with its trafficking across the surface of EC [64,65]. Moreover, sVEGFR-1 is reported to directly downregulate the expression and signaling of VEGFR-2 [64].

The activation of VEGFR-2 by VEGF is associated with autophosphorylation of at least five tyrosine residues which in turn promote pro-angiogenic responses that are effective via their respective pathways [66]. Ahmad et al. reported that sVEGFR-1 upregulation in EC abrogated Y951 phosphorylation site [67]. Additionally, Takahashi et al. showed a significant downregulation of VEGF-dependent cell proliferation when the VEGFR-2 signaling pathway was blocked [40].

VEGF-A-stimulated activation of VEGFR-2 is significantly attenuated in hypoxic environments such as that of PE [68]. Downregulation of VEGFR-2 could impair the activation of pathways such as the PI3K/Akt pathways which may promote oxidative stress through decreasing eNOS and NO. Vasodilatory, anti-inflammatory, and anti-thrombotic effects are associated with NO bioavailability [69]. Hence, decreased VEGFR-2 may contribute to both, the anti-angiogenic and pro-inflammatory state of PE. Similar to VEGFR-2, the expression of 2-Methoxyestradiol (2-ME) is reportedly decreased in PE [70]. Lee and Nevo reported that PE-like induced animal models increased expression levels of VEGFR-2 under hypoxic conditions when treated with 2-ME [71]. However, the mechanism of action by which 2-ME increases the expression of VEGFR-2 is yet to be elucidated [71]. Earlier research has highlighted HIV infection and the use of cART as risk factors for the development of PE due to their exacerbation of the anti-angiogenic and pro-inflammatory environment of PE [6,72].

## 5. Human Immunodeficiency Virus

Human Immunodeficiency Virus infection is a global concern with 38.4 million people living with HIV at the end of 2021 [73]. According to a 2021 UNAIDS report, there were 1.5 million new infections worldwide [73]. In the same year, 20.6 million individuals in Eastern and Southern Africa were infected with HIV, with almost 16.2 million receiving cART [5]. South Africa encountered 670,000 new infections in 2021 and has the largest ARV rollout in the world with approximately 90% of pregnant South African women receiving cART for the prevention of mother-to-child transmission [74].

The World Health Organization (WHO) has recommended that all HIV-positive individuals begin and continue using cART [75]. Due to its potential to prevent vertical transmission, pregnant and breast-feeding women are encouraged to continue with cART treatment [75]. It is widely accepted that cART stimulates immune reconstitution and a hyperinflammatory response [76]. However, there is controversy as to whether HIV treatment impacts the clinical management of PE [77].

HIV infection causes a decrease in CD4 T cells, indicating immunosuppression [78]. Several studies have postulated that HIV infection influences the rate of PE development [79,80,81]. Since a heightened immunological response is characteristic of PE, HIV infection should theoretically lower the risk of developing PE; however, reports are conflicting [81,82]. Nevertheless, many recent studies have reported an increase in the risk of developing PE in HIV-positive patients receiving cART [6].

## 6. The Role of VEGFR-2 in HIV-Associated Preeclampsia

HIV-1 encodes at least nine genes in its RNA genome, including the trans-activator of transcription (tat) protein [83]. The tat accessory protein is released from HIV-infected cells and significantly enhances the efficiency of viral transcription [83]. It has the ability to bind to and interact with receptors on the surface of EC [84].

**Figure 2:**
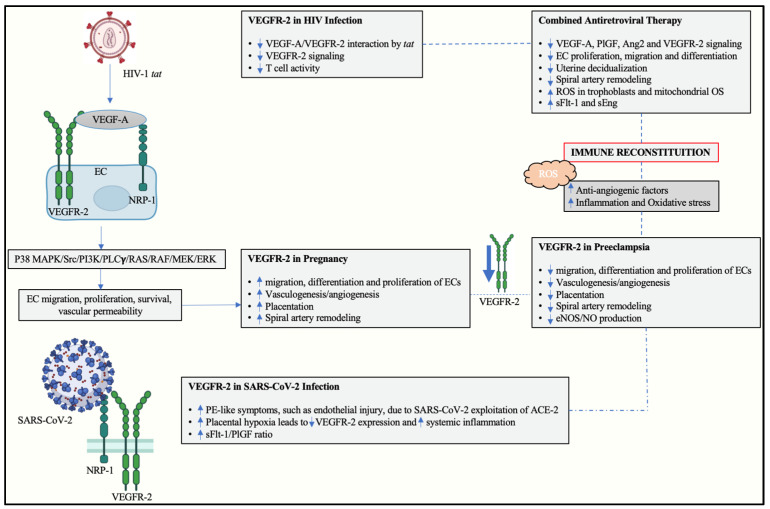
VEGFR-2 regulation in PE, HIV and COVID-19. Abbreviations: vascular endothelial growth factor receptor-2 (VEGFR-2), vascular endothelial growth factor-A (VEGF-A), neuropilin-1 (NRP-1), Severe Acute Respiratory Syndrome Coronavirus 2 (SARS-CoV-2), Angiotensin converting enzyme-2 (ACE-2), Soluble fms-like tyrosine kinase-1 (sFlt-1), placental growth factor (PlGF), angiotensin-2 (Ang-2), Reactive Oxygen Species (ROS), oxidative stress (OS), endothelial cell (EC), Mitogen-activated protein kinase (MAPK), phospholipase-Cγ (PLCγ), protein kinase C (PKC), Rat sarcoma virus (RAS), Rapidly Accelerated Fibrosarcoma (RAF), Extracellular-signal-regulated kinase (ERK), Phosphoinositide-3 kinase (PI3K), protein kinase B (Akt), endothelial nitric oxide synthase (eNOS), nitric oxide (NO).

The tat protein of HIV-1 possesses an arginine- and lysine-rich sequence similar to several other growth factors, including fibroblast growth factor, VEGF-A, hepatocyte growth factor, and heparin-binding epidermal growth factor [85,86]. The similarity in the sequence enables tat to mimic the angiogenic effects of VEGF by binding to and activating VEGFR-2 (Figure 2). This protein binds to VEGFR-2 with a similar affinity to the receptor’s endogenous ligand [85]. Previous studies reported tat-stimulated EC growth and migration in vitro, as well as tat-induced angiogenesis in vivo [85,87]. Interestingly, the binding of tat to VEGFR-2 is specific as no interaction occurs between the tat protein and a range of other tyrosine kinase receptors [85]. This highlights a possible therapeutic potential in targeting of the tat-VEGFR-2 interaction; however, this requires further investigating. Furthermore, chronic HIV infection is associated with chronic arterial injury and subsequent EC damage [88]. Therefore, it is plausible to assume that chronic HIV infection affects EC competence to produce VEGFR-2.

Moreover, Gavalas et al. demonstrated an inhibitory effect of VEGF-A, via VEGFR-2, on the proliferation and cytotoxic activity of T cells from ascites of ovarian cancer patients [89]. Although this investigation was performed in cancer patients, it may be plausible that the VEGF-A-mimicry effect of tat may cause a similar inhibitory effect upon binding with VEGFR-2. This may lead to increased viral load and dissemination due to the inhibition of T cells.

Notably, it is possible that HIV infection neutralizes the immune response in PE while the effects of the tat protein would promote angiogenesis; however, neither scenario occurs in treated people. Reportedly, cART in pregnancy reconstitutes the immune response and predisposes PE development; however, the effect of the duration of cART on immune restoration requires investigation [90]. Furthermore, the non-realization of the angiogenic effects of the tat protein is attributed to the use of cART and its potent effect in reducing and maintaining viral load. However, the lack of research investigating VEGFR-2 in HIV infection highlights the urgent need for future research evaluating the regulation of VEGFR-2 in viral infections and the effect of treatment.

## 7. SARS-CoV-2 Infection and the Influence of VEGF and Its Receptors

The novel SARS-CoV-2 emerged in late November–December 2019 and led to the Coronavirus Disease-2019 (COVID-19) pandemic [91]. Approximately 600 million infections have been reported worldwide with almost 7 million deaths [92]. South Africa has reported over 4 million infections and more than 100,000 deaths [92]. Sheffield reported that pregnant women infected with SARS-CoV-2 are more likely to require hospitalization and ICU admission compared to non-pregnant women; therefore, COVID-19-positive pregnant women are more at risk of encountering adverse outcomes [93]. However, the COVID-19 vaccine rollout has significantly reduced disease severity in the general population, including pregnant women [94,95]. Furthermore, two studies reported adverse maternal and neonatal outcomes with SARS-CoV-2 infection in pregnancy in South African cohorts; this was further confirmed through a multinational investigation [96,97,98]. Approximately 5 billion individuals have been vaccinated globally with observational studies noting no concerns of short-term adverse outcomes following vaccination in pregnant women [92,99,100]. Passive immunity may be passed down from mother-to-child through breast milk since it is high in SARS-CoV-2-specific IgA and IgG [101]. Although the COVID-19 vaccine has shown high efficacy in protecting against severe SARS-CoV-2 infection, long-term evaluation is essential to determine the possible side effects associated with the COVID-19 vaccine [99].

Infection by SARS-CoV-2 and the development of COVID-19 is associated with endothelial dysfunction and inflammation [102]. Upon viral entry into the host, SARS-CoV-2 attaches to angiotensin-converting enzyme 2 (ACE 2) and NRP-1 receptors of pneumocytes, thereby infecting host cells [103]. An upregulation of E-selectin, P-selectin, adherins, cadherins, VEGF-A, NRP-1, VEGFR-1, VEGFR-2, PECAMs, ICAMs, VCAM-1, and hypoxia has been reported in SARS-CoV-2 infected lung compared to control tissue [17,102,104,105]. Dysregulation of these proteins results in increased vascular permeability. Furthermore, the VEGF-A/VEGFR-2 axis recruits the TSAd (T cell specific adapter) protein complex which is responsible for the regulation of VEGF-A-induced activation of Src (proto-oncogene tyrosine-protein kinase sarcoma) tyrosine kinase that influences vascular permeability in EC [22]. The recruitment of the TSAd protein complex ultimately results in increased cell proliferation, migration, and survival as well as increased EC permeability [22]. It is plausible that increased cell permeability through the TSAd protein complex may be responsible for the pulmonary edema that occurs in severe COVID-19. Hence, targeting the VEGF/VEGFR-2 signaling pathway may serve as a potential therapeutic strategy for treating COVID-19 [106].

Moreover, the endothelial pathology identified in COVID-19-positive individuals resemble the pro-inflammatory state of PE [10]. Placentae from COVID-19-positive patients showed increased placental hypoxia with a consequential decline in maternal vascular perfusion indicating systemic inflammation [107]. Notably, placental hypoxia is associated with the activation of a pro-inflammatory response and a decrease in VEGFR-2 expression, favoring an anti-angiogenic imbalance through pathways alluded to earlier [55,56]. Additionally, an increase in the sFlt-1/PlGF ratio was observed in COVID-19-positive compared to healthy patients; this upregulation is also evident in PE [108]. The sFlt-1/PlGF ratio is a powerful diagnostic tool that is currently used in the early diagnosis and management of PE worldwide [109]. Thus, it is plausible that SARS-CoV-2 infection may heighten the risk of developing PE while PE may increase the risk of developing severe COVID-19 [57,110].

Dysregulation of VEGFR-2 is associated with PE, HIV infection, and SARS-CoV-2 infection. The pathogenesis of PE and the effect of HIV infection, cART usage, and SARS-CoV-2 infection are explored in Figure 2.

## 8. The Involvement of VEGFR-2-Specific MicroRNAs in Preeclampsia, HIV and SARS-CoV-2 Infection

**Figure 3:**
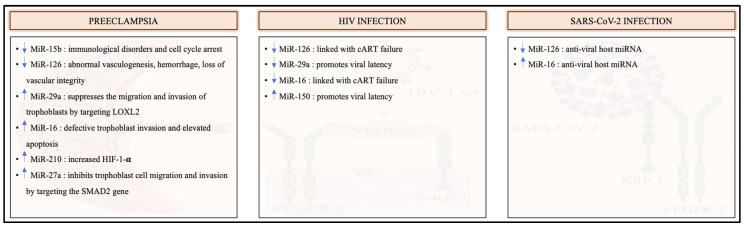
MicroRNA regulation in PE, HIV, and COVID-19. Abbreviations: lysyl oxidase homolog 2 (LOXL2), hypoxia inducing factor-1-α (HIF-1-α), combined antiretroviral therapy (cART).

MicroRNAs have been implicated in the progression of normal physiological processes such as pregnancy, as well as in pathological conditions such as cardiovascular diseases, cancer, viral infections, and PE. MicroRNAs post-transcriptionally regulate the expression of VEGFR-2 in normal and pathological conditions [18,111]. Figure 3 explores microRNA regulation of VEGFR-2 in PE, HIV, and SARS-CoV-2 infection.

MiR-15b, miR-126, miR-29a, and miR-16 are dysregulated in PE, HIV infection, and COVID-19 and influence VEGFR-2 mRNA both directly and indirectly (Table 1) [18,19,111,112,113,114]. It was reported that miR-15b is downregulated in PE and HIV but upregulated in COVID-19 [18,112]. MiR-15b is implicated in immunological disorders and cell cycle arrest by targeting cyclin E1 in cancer cell lines [115]. Earlier studies have highlighted the downregulation of miR-126 in PE, HIV infection, and COVID-19 [18,19]. Inhibition of miR-126 in a mouse model causes abnormal vasculogenesis, hemorrhage, and loss of vascular integrity [116]. Additionally, there is a downregulation of miR-126 in HIV-positive patients receiving cART; however, miR-126 is upregulated in patients with cART resistance, indicating a link with treatment failure [18]. The nucleocapsid of SARS-CoV-2 is a target of miR-126, making miR-126 an anti-viral host miRNA [117]. MiR-29a was reported to be upregulated in PE but downregulated in HIV infection and COVID-19 [111,113]. Xu et al. reported that the upregulation of miR-29a in PE suppresses the migration and invasion of trophoblasts by directly targeting the LOXL2 (lysyl oxidase homolog 2) mRNA; hence, showing a negative correlation between miR-29a and trophoblastic migration and invasion [111]. Notably, the *nef* gene of HIV-1 is a target of miR-29a. Houzet et al. observed a decrease in the nef mRNA and viral load that positively correlated with miR-29a suppression [118]. The decrease in miR-29a in HIV could be a compensatory mechanism to maintain the latent state of infection [11,119]. MiR-16 is upregulated in PE and COVID-19 but downregulated in HIV infection [18,114]. Yuan et al. reported that an upregulation of miR-16 inhibits proliferation, migration, and invasion, and promotes apoptosis in JEG-3 cell lines through the inhibition of Notch2 (neurogenic locus notch homolog 2) [114]. This implicates miR-16 in both defective trophoblast invasion and its elevated apoptosis in the pathophysiology of PE. Similar to miR-126, miR-16 is downregulated in HIV-positive patients who are resistant to cART [18]. Nersisyan et al. found miR-16 to be a potential host miRNA that can bind coronavirus through an in silico investigation [120].

Commonly dysregulated in PE and HIV infection, miR-210, miR-150, miR-122, and miR-27a regulate the expression of VEGFR-2 (Table 2) [121,122,123,124,125,126,127,128,129]. Lasabova et al. reported a significant increase in miR-122 in preeclamptic compared to control placentae [126]. MiR-122 exhibited apoptotic regulatory properties and was reported to promote apoptosis following the suppression of miR-122 in cancer [115]. Moghoofei et al. reported an upregulation of miR-122 in HIV-positive patients, with a positive correlation between the expression of miR-122 and HIV viral load [127]. Bounds et al. reported an increase in miR-210 in PE [121]. MiR-210 is upregulated during hypoxic conditions; however, HIF-1-α, causes a positive feedback loop since it stimulates the expression of miR-210 [130]. Ballegaard et al. reported an upregulation of miR-210 in HIV-positive PBMCs compared to uninfected PBMCs [123]. Notably, miR-150 is upregulated in both PE and HIV infection [124,125]. Huang et al. reported that inhibition of miR-150 can stimulate virus production in HIV-positive patients receiving cART [131]. Hence, upregulation of miR-150 may promote viral latency in HIV infection. Furthermore, miR-27a is upregulated in PE and HIV infection [128,129]. Zheng et al. reported that upregulated miR-27a in PE targets the SMAD2 gene, thus inhibiting trophoblast cell migration and invasion [128]. Additionally, in the event of an upregulation of miR-126, miR-122, and miR-27a, the expression of VEGFR-2 is heightened [115,132,133]. In contrast, an upregulation of miR-15b, miR-29a, miR-16, miR-210, and miR-150 results in the downregulation of VEGFR-2 [134,135,136,137,138].

Although differentially expressed in PE, HIV infection, and COVID-19, miRNAs have diverse roles in pathological conditions. The relationship between these specific miRNAs and VEGFR-2 is summarized in Table 3.

## 9. Conclusions

The theoretical framework underpinning VEGF/VEGFR-2 interaction suggests the fundamental role of VEGFR-2 in angiogenic signaling in various pathological conditions including PE, HIV infection, and SARS-CoV-2 infection. The impasse of the VEGFR-2 signaling pathway in animals leads to a significant decline in EC proliferation. Similarly, alterations to the genes encoding VEGF receptors promote embryonic death as a consequence of abnormal blood vessel formation. In humans, the expression of VEGFR-2 is downregulated in PE while it is upregulated in both HIV and SARS-CoV-2 infection. Additionally, modulation of miRNAs through antagomirs (antagonistic miRNAs) serves as a possible novel therapeutic agent due to its influence on VEGFR-2 mRNA action.

## 10. Future Recommendations

Future investigations are needed to identify the impact of HIV infection on the expression levels of VEGFR-2. There is an abundance of in silico investigations conducted with respect to COVID-19; in accordance, there is a dire need for animal studies to investigate further and confirm these results. We recommend that further research is performed to completely elucidate the effects of cART in the development of PE. Moreover, the influence of miRNA regulation in PE, HIV, and SARS-CoV-2 infection should be investigated in a large cohort to rectify inconsistencies in the literature.

## Figures and Tables

**Table 1:** The expression of VEGFR-2-related microRNAs commonly dysregulated in PE, HIV infection, and COVID-19.

MicroRNA	PE	HIV	COVID-19	References
miR-15b	Downregulated	Downregulated	Upregulated	[18,112]
miR-126	Downregulated	Downregulated	Downregulated	[18,19]
miR-29a	Upregulated	Downregulated	Downregulated	[111,113]
miR-16	Upregulated	Downregulated	Upregulated	[18,114]

**Table 2:** The expression of VEGFR-2-related microRNAs commonly dysregulated in PE and HIV.

MicroRNA	PE	HIV	References
miR-210	Upregulated	Upregulated	[121,122,123]
miR-150	Upregulated	Upregulation	[124,125]
miR-122	Upregulated	Upregulated	[126,127]
miR-27a	Upregulated	Upregulated	[128,129]

**Table 3:** The expression of VEGFR-2 in the event of miRNA upregulation.

MicroRNA	VEGFR-2 Expression	References
miR-15b	Downregulation	[134]
miR-126	Upregulation	[132]
miR-29a	Downregulation	[135]
miR-122	Upregulation	[115]
miR-27a	Upregulation	[133]
miR-16	Downregulation	[136]
miR-210	Downregulation	[137]
miR-150	Downregulation	[138]

## Data Availability

Not applicable.
